# The Past, Present and Future of Tuberculosis

**Published:** 1907-09

**Authors:** 


### THE PAST, PRESENT, AND FUTURE OF TUBERCULOSIS.

Shattuck observes that in the State of Massachusetts the work in the treatment of tuberculosis for the near future would seem to lie in: The passage of a general law making the reporting of consumption compulsory. But no such law can be thoroughly efficient unless the medical profession as a whole is competent to make an early diagnosis, and is in such sympathy with the law that the physician puts obedience to it and its underlying principle above the selfish preference of this or that patient and above any fear that he may lose practice by reporting him. It is not likely that such opposition will be very serious or long lived. Laws providing for the disinfection of dwellings occupied by the comsumptive after moving or death. An extension of the antispitting law so as to include at least factories, mills, schools and the halls of tenement houses. Legislative provision for State medical inspectors, responsible to the State Board of Health, each in charge of a district within which he supervises organized work. Eeach community in the organization should have such force of nurses, vistors, or both, as each may need; that the poor may be followed up in their homes, taught what to do, help to do it, if necessary, and encouraged to persevere in doing it. Much of this can be done without, or with trifling expense. There is much intelligent energy running to waste which can be turned in this direction. —Boston Med. & Surg. Jour., Aug. 1, 1907.

